# Sarcopenia is Associated with Malnutrition but Not with Systemic Inflammation in Older Persons with Advanced CKD

**DOI:** 10.3390/nu11061378

**Published:** 2019-06-19

**Authors:** Simone Vettoretti, Lara Caldiroli, Silvia Armelloni, Camilla Ferrari, Matteo Cesari, Piergiorgio Messa

**Affiliations:** 1 Unit of Nephrology, Dialysis and Renal Transplantation—Fondazione IRCCS Ca’Granda Ospedale Maggiore Policlinico di Milano, 20122 Milan, Italy; simone.vettoretti@policlinico.mi.it (S.V.); lara.caldiroli@policlinico.mi.it (L.C.); silvia.armelloni@policlinico.mi.it (S.A.); camilla.ferrari4@studenti.unimi.it (C.F.); 2 Unit of Geriatrics—Fondazione IRCCS Ca’ Granda Ospedale Maggiore Policlinico di Milano, 20122 Milan, Italy; matteo.cesari@policlinico.mi.it; 3 Department of Clinical Sciences and Community Health, University of Milan, 20122 Milan, Italy

**Keywords:** sarcopenia, chronic kidney disease, malnutrition, inflammation, physical performance

## Abstract

Background: In patients with chronic kidney disease (CKD), sarcopenia can be determined by a wide spectrum of risk factors. We evaluated the association of sarcopenia with nutritional, behavioral and inflammatory patterns in older patients with advanced CKD. Methods: we cross-sectionally evaluated 113 patients with CKD stages 3b-5. Sarcopenia was defined according to the EWGSOP2 criteria. We assessed: anthropometry, bioelectrical impedance analysis, physical, and psychological performance. Nutritional status was assessed using the Malnutrition Inflammation Score (MIS) and by verifying the eventual presence Protein Energy Wasting syndrome (PEW). Systemic inflammation was assessed by dosing: CRP, IL6, TNFα, MCP1, IL10, IL17, fetuin, IL12. Results: 24% of patients were sarcopenic. Sarcopenic individuals had lower creatinine clearance (18 ± 11 vs. 23 ± 19 mL/min; *p* = 0.0087) as well as lower BMI (24.8 ± 3.0 vs. 28.4 ± 5.5 Kg/m^2^; *p* < 0.0001) and a lower FTI (11.6 ± 3.9 vs. 14.4 ± 5.1 kg/m^2^, *p* = 0.023). Sarcopenic persons had higher prevalence of PEW (52 vs. 20%, *p* < 0.0001) and a tendency to have higher MIS (6.6 ± 6.5 vs. 4.5 ± 4.0, *p* = 0.09); however, they did not show any difference in systemic inflammation compared to non-sarcopenic individuals. Conclusions: CKD sarcopenic patients were more malnourished than non-sarcopenic ones, but the two groups did not show any difference in systemic inflammation.

## 1. Introduction

Sarcopenia is a clinical condition characterized by the reduction of skeletal muscle strength and low muscle quantity or quality. The evaluation of physical function contributes to the definition of the severity of sarcopenia [1].

Sarcopenia has to be kept distinct form cachexia which is a multifactorial syndrome characterized by progressive loss of muscle mass (with or without loss of fat mass) [2]. In cachexia, the main diagnostic criteria are weight loss or low BMI while reduced muscle strength is just an additional criterium together with reduced fat mass, fatigue, anorexia, low fat-free mass, and abnormal biochemistry [3].

In patients with chronic kidney disease (CKD), the prevalence of sarcopenia ranges from 5% to 37%, depending on the adopted definition and on the stage of renal disease [4,5].

In CKD patients, regardless of the initiating factor, the loss of muscle mass depends on a negative balance of protein homeostasis. Furthermore, CKD is associated with impaired regeneration of muscular tissue (cell structural changes, reduced cell activation and satellite cell inactivation) [2].

The pathophysiology of sarcopenia in CKD is multifaceted and depends on: muscular, systemic and behavioral causes [6]. The principal cause of muscle wasting is the activation of the ubiquitin-proteasome system (UPS). Both inflammation (CRP, IL6 and TNFα) and metabolic acidosis trigger the activation of ATP-UPS. TNFα triggers protein degradation also by activating the NFκB pathway. Metabolic acidosis occurs mainly thought a strong accumulation of the uremic toxin indoxil sulphate (IS), which on one hand activates the UPS and on the other hand induces oxidative stress and metabolic changes of muscle cells. CKD patients have high levels of angiotensin II which activates the caspase-3 pathway and reduces circulating and skeletal IGF-1. Both these mechanisms are associated with muscle protein wasting and apoptosis of muscle cells. Both insulin levels and insulin resistance may contribute to the development of sarcopenia in CKD patients since both these factors activate the PI3K pathway that is associated with protein wasting and decreased protein synthesis.

In CKD patients, muscle regeneration and size is also affected by the accumulation of myostatin, a negative regulator of skeletal muscle mass [7]. Finally, sarcopenia is also associated to reduced appetite, physical inactivity and comorbidities [8]. In the general population, sarcopenia is associated with negative outcomes such as physical disability, poor quality of life and mortality [9,10,11,12]. Pre-dialysis CKD patients affected by sarcopenia have an overall increase of cardiovascular risk [13] and mortality [4]. Furthermore, in CKD patients, a decrease in muscular strength is associated with the composite outcome of progression to end-stage renal disease and mortality [14].

In this study, we evaluated the association of sarcopenia with several modifiable (nutritional and behavioral) and not modifiable (systemic inflammation) risk factors in elderly subjects with advanced CKD not yet on dialysis.

## 2. Materials and Methods

### 2.1. Patients Characteristics

We cross-sectionally evaluated 113 prevalent patients attending our outpatient clinic. We applied the following selection criteria: all patients were ≥65 years old, with CKD stages 3b to 5 (10 ≤ eGFR ≤ 45 mL/min/m^2^) in conservative therapy. Glomerular filtration rate (eGFR) was estimated according to the CKD-EPI formula [15]. All patients included in the study had to have a relatively stable eGFR over the previous 6 months (with less than 2 mL/min of variation).

In order to exclude possible confounding factors, we considered the following excluding criteria: active cancer; decompensated chronic liver diseases (advanced cirrhosis and/or ascites); severe heart failure (NYHA class III—IV); nephrotic syndrome; depression; hypo- or hyperthyroidism; malabsorption diseases; and inability to cooperate. We also excluded patients who were treated with immunosuppressive drugs or had experienced a recent hospitalization (during the past six months).

In order to explore systemic inflammatory status, CKD patients were compared with 15 healthy controls with preserved eGFR (average eGFR 79 ± 5 mL/min/1.73 m^2^) and normal urine examination that were matched for age. All controls had to fulfill the same inclusion and exclusion criteria that were applied to CKD patients.

The study was conformed to the ICP Good Clinical Practices Guidelines and to the declaration of Helsinki, and it was approved by the Ethics Committee of our Institution (approved 23 October 2010 doc 347/2010). All patients signed an informed consent to participate.

Medical history, nutritional status, physical performance tests, and cognitive and psychological assessment were performed in a unique visit. Urinary and biochemical parameters were collected on the same visit, in the morning and after an overnight fast of at least 12 h. Alimentary diaries were compiled during the 2 days preceding the visit.

### 2.2. Assessment of Sarcopenia

Sarcopenia was defined, according to the European Working Group on Sarcopenia in Older People (EWGSOP2) criteria, as the coexistence of low muscle strength and reduced muscle quantity [16].

Muscle strength was evaluated by handgrip strength using a Jamar hand dynamometer (Sammons Preston Inc., Bolingbrook, IL, USA). Handgrip strength was considered reduced for values <16 kg in females and <27 kg in males [16].

Reduced muscle mass was defined as a reduction of mid-arm muscle circumference (MAMC) > 10% in relation to the 50th percentile of the reference population [16].

In order to evaluate the severity of sarcopenia, we determined the proportion of patients with reduced speed (<0.8 m/s) at the 4 m gait speed test.

### 2.3. Nutritional Intake, Body Composition and Nutritional Status

Energy (Kcal/kg/day) and macro-nutrients intakes were estimated from the average of two dietary diaries (food-intake record) that were compiled in the 2 days preceding the visit. Protein intake was also estimated by determining normalized protein catabolic rate (nPCR) on the 24 h urinary urea excretion [17].

Anthropometric measurements included: body weight, height, body mass index (BMI, calculated according to Quetelet Index (kg/m^2^)), waist circumference (WC); tricipital and bicipital skinfold (TST; BST), measured with a Harpenden skinfold caliper, as well as mid-arm circumference (MAC) and calf circumference (CC). MAMC was derived from MAC and TST as follows: MAMC (cm) = AC (cm) − (πxTSF (cm)); these measurements were performed on the dominant arm.

Body composition was measured by using a multifrequency bioelectrical impedance analysis device (Body Composition Monitor, Fresenius Medical Care, Bad Homburg, Germany) which determined: lean tissue index (LTI), fat tissue index (FTI), adipose tissue mass (ATM), fat tissue (FT), lean tissue mass (LTM), and body cell mass (BCM).

Nutritional status was assessed by evaluating the presence of protein energy wasting syndrome (PEW) and by determining the individual malnutrition inflammation score (MIS). PEW was assessed using the criteria defined by the International Society of Renal Nutrition and Metabolism [18]. In particular, PEW was defined by the presence of one item in at least three of the four domains for malnutrition assessment [19]. Malnutrition-inflammation score (MIS) is a validated scoring system for the assessment of malnutrition and inflammation in patients with CKD [20]. A total score of 4–7 was considered indicative of mild malnutrition and a score ≥8 of severe malnourishment [21].

### 2.4. Physical Performance

Short physical performance battery (SPPB) includes a hierarchical test of standing balance, a 4-m walk and five repetitive chair-stands [22]. Each SPPB component test is scored from 0 to 4 with a score of 0 representing the inability to perform the test and a score of 4 representing the highest category of performance [23].

The Lawton’s scale regarding the instrumental activities of daily life (IADL) considers the evaluation of eight items: use of telephone (A), shopping (B), food preparation (C), housekeeping (D), laundry (E), mode of transportation (F), medication (G), and finances (H) [24]. Scoring range is 0–8 in women (all items), and 0–5 in men (except for items C, D and E) [25].

Physical Activity Scale (PAS) is a brief and easily-scored survey test that was specifically designed to assess physical activity of people aged ≥65 years. It combines information on leisure, household and occupational activities. The scale consists of five domains (walking, taking the stairs, housekeeping, shopping, additional physical activity) that are scored 0 to 3 each; thus, very active people have a score >12 and completely inactive ones a score of 0 to 3.

### 2.5. Assessment of Depression

The Geriatric Depression Scale (GDS) is based on a self-filled quiz form, and it assesses the presence and severity of depression in older adults. GDS is developed as a 30-item instrument and users respond in a “Yes/No” format; a score ≥11 is considered indicative of depression [26].

### 2.6. Assessment of Frailty

Frailty was assessed by using the frailty phenotype proposed by Fried and colleagues. The presence of three or more of the following characteristics support the presence of frailty: unintentional weight loss, exhaustion, weakness, slow gait speed, and low physical activity [18].

### 2.7. Biochemical Parameters

All biochemical analyses (in serum and urines) for the evaluation of renal function and metabolic and nutritional status were performed at the central laboratory of our Institution on the same day of the visit.

### 2.8. Detection of Serum Levels of Cytokines

Serum samples for the determination of cytokines concentration were collected on the day of the visit, then they were frozen and stored at −80 °C. All samples were thawed at most three times. Cytokines concentrations were performed by using enzyme-linked immunosorbent assay (ELISA) kits following the manufacturer’s instructions.

The following kits were used: Human IL-10 ELISA Kit EHIL10 (Invitrogen, Thermo Fisher Scientific, Monza, Italy), Quantikine ELISA Human CCL2/MCP-1 Immunoassay DCP00, Quantikine ELISA Human IL-12 p70 Immunoassay D1200 (all R&D Systems, Space, Milano, Italy) Human TNF-alpha ELISA Kit ((Thermo Fisher Scientific, Monza, Italy), Human Fetuin-A D191037100 (Li StarFish S.r.l., Cernusco S/N, Italy). Declared kit sensitivity was <3 pg/mL for IL-10, 1.7 pg/mL for CCL2/MCP-1, 5 pg/mL for IL-12p70, <2 pg/mL for TNFalpha, 0.104 ng/mL for Fetuin-A. For the quantification of IL-17, Quantikine ELISA Human IL-17 Immunoassay D1700 kit was used, extending the curve dilution to 3.9 pg/mL, given that the serum values of controls indicated in the literature (Zhu X. 2018) were very low. For IL-6 dosage, three different ELISA kits, with standard curve ranges of decreasing values, were used and compared: Human IL-6 ELISA Kit EH2IL6 (Thermo Fisher Scientific, Monza, Italy), Human IL-6 Platinum ELISA BMS213/2 (Affymetrix, Thermo Fisher Scientific, Monza, Italy) and Quantikine HS ELISA Human IL-6 Immunoassay HS600B (R&D Systems, Space, Milano, Italy), with sensitivity of <1 pg/m, 0.92 pg/mL and 0.110 pg/mL respectively. For IL-6 quantification, Quantikine HS ELISA, Human IL-6 Immunoassay HS600B and Human IL-6 ELISA Kit EH2IL6 results were compared by a simple regression test and both results were indifferently used after ascertaining the significant correlation.

In each test, the curve included the zero as the last standard point. Quantikine Immunoassay Control Group 1–4 or 10 (R&D Systems, Space, Milano, Italy), as appropriate, were used to check the acceptability of the assays. Absorbance readings were measured at 450 nm by spectrophotometer (Xenius Safas, Monaco). All cytokines values were evaluated in duplicate.

### 2.9. Statistical Analysis

All data are expressed as mean ± SD or median ± IQR as appropriate. The comparison of parametric variables was done using Student’s *t*-test while the comparison of not parametric ones was done using the Mann-Whitney “*U*” test. Proportions and categorical variables were compared using the independent chi-squared (χ^2^) test or the Fisher’s exact test. Statistical analysis was carried out with Statview software version 5.0.1.

## 3. Results

### 3.1. Cohort Characteristics

Patients’ characteristics are reported in Table 1. Mean age was 80 ± 6; 68% of patients were male and 54% had diabetes. The prevalence of sarcopenia was 24% (27/113). Sarcopenic patients had lower creatinine clearance. Estimated GFR was lower in sarcopenic individuals but it did not reach statistical difference. All sarcopenic patients had reduced handgrip strength, reduced MAMC and higher prevalence of impaired gait speed. Sarcopenic patients totalized worse scores at all physical performance tests: SPPB (6.0 ± 3.8 vs. 8.0 ± 4.0, *p* = 0.0008), PAS (4 ± 6 vs. 7 ± 5, *p* = 0.0014) and IADL scale (5.0 ± 1.8 vs. 5.0 ± 1.0, *p* = 0.016). The two groups of patients had the same proportion of frail and depressed individuals. However, sarcopenic ones had higher average depression scores (11.8 ± 7.1 vs. 8.3 ± 5.5; *p* = 0.008).

### 3.2. Nutritional Parameters

Nutritional parameters are shown in Table 2 and Table 3. Non-sarcopenic patients were more often prescribed a hypoproteic diet (38 vs. 11%; *p* = 0.0065, Table 2). We did not find any difference in serum concentrations of biochemical nutritional parameters between sarcopenic and non-sarcopenic individuals (Table 2). Vitamin D status as well as vitamin D supplementation rate were not different in sarcopenic and non-sarcopenic individuals (Table 2). Sarcopenic patients had lower values of urinary urea and creatinine excretions and urinary phosphorous (13,600 ± 6022 vs. 16,400 ± 8832 mg/24 h, *p* = 0.02, 713 ± 274 vs. 920 ± 320 mg/24 h, *p* = 0.0034 and 381 ± 146 vs. 512 ± 197 mg/24 h, *p* = 0.0025 respectively). Nevertheless, the nPCR was not different in the two subgroups (Table 2). The analysis of dietary diaries (Table 2) revealed higher estimated intake of calories, normalized per body mass, in sarcopenic patients (24 ± 8 vs. 20 ± 9 Kcal/Kg¸ *p* = 0.0058), with no differences in the intake of macronutrients between the two groups (Table 2). In Table 3, we summarized the parameters referring to body composition and nutritional scores. Sarcopenic individuals had lower BMI (24.8 ± 3.0 vs. 28.4 ± 5.5 Kg/m^2^; *p* < 0.0001) and a lower FTI (11.6 ± 3.9 vs. 14.4 ± 5.1 kg/m^2^, *p* = 0.023). Sarcopenic patients tended to have a higher MIS (6.6 ± 6.5 vs. 4.5 ± 4.0, *p* = 0.09) as well as higher prevalence of PEW (52 vs. 20%, *p* = 0.0008). We also evaluated whether the singular criteria of PEW (according to ISRNM definition) were differently distributed between the two groups (Table 3), but we did not find any differences between the two groups.

### 3.3. Inflammation and Sarcopenia

Systemic inflammatory status was assessed in 109 of the 113 CKD patients and in 15 healthy controls (Table 1). Comparing CKD patients with healthy controls, we found that CKD individuals had higher levels of: TNFα 14.6 (2.4–42.5) vs. 7.1 (1.5–20.5) pg/mL (*p* = 0.014); IL-12p70 0.7 (0–19) vs. 0 (0–1.7) pg/mL (*p* = 0.002) and MCP-1 407 (21–886) vs. 254 (199–486) pg/mL (*p* = 0.002), as well as lower concentrations of Fetuin-A 0.31 (0.12–0.76) vs. 0.41 (0.18–0.84) g/L (*p* = 0.010). The two groups did not show any significant difference regarding: IL-6 3.4 (0–12.9) vs. 1.1 (0–17.3) pg/mL (*p* = 0.066); IL-10 2 (0–740) vs. 5 (1–18) pg/mL (*p* = 0.464) and IL-17 1.5 (0–17.6) vs. 1.9 (0.5–6.8) pg/mL (*p* = 0.314) (Table 4). We evaluated also whether there was any difference in the inflammatory status of sarcopenic and non-sarcopenic CKD patients; however, we did not find any difference in the concentration of inflammatory cytokines between the two groups (Table 5).

## 4. Discussion

Our study indicates that among the older patients with advanced CKD, sarcopenia is highly prevalent (24%). Sarcopenic subjects had lower BMI, higher prevalence of PEW, and higher average MIS. Although we did not observe any difference in the estimated intake of macro-nutrients between the two groups, sarcopenics were found to assume a higher quantity of calories (normalized per body mass). Sarcopenics had impaired physical performance, overall reduced physical activity and resulted to be more depressed.

Serum concentrations of several inflammatory cytokines were higher in CKD individuals than in healthy controls. However, among CKD individuals, sarcopenic did not show any difference in cytokines concentration with respect to non-sarcopenicones.

The prevalence of sarcopenia in CKD patients is extremely variable and depends on the definition that is adopted and on the stage of renal disease; until now, there is not a universally recognized definition of sarcopenia in CKD [5]. De Souza and co-authors [5] analyzed a cohort of patients with advanced CKD not yet on dialysis and reported a prevalence of sarcopenia that was 11.9% and 28.7% by using, respectively, the definitions of the Foundation for the National Institute of Health (FNIH) [5] and of the European Working Group on Sarcopenia in Older People (EWGSOP) [27]. In a cohort of CKD patients that included subjects in conservative therapy (*n* = 26), on dialysis (*n* = 37) and with a kidney transplant (*n* = 14), Lai and colleagues reported a prevalence of sarcopenia that was 49.4% [13]. Our results confirm these previous results. The higher prevalence of sarcopenia that was reported by Lai et al. probably depends on the fact that they included in their analysis also subjects that were already on dialysis, a condition that is associated with a faster reduction of muscle mass and function [2].

Independently of the definition that is adopted, sarcopenia is invariably associated with worse clinical outcomes [2]. Whether this is related to sarcopenia per se or is dependent on the concomitant PEW has not been established yet. This aspect is further complicated by the fact that muscle wasting is one of the criteria considered for the definition of PEW syndrome [18]. In our cohort, sarcopenic patients had lower BMI and reduced fat mass in respect to non-sarcopenic ones. Thus, it may be that malnutrition played a pivotal role in the onset of sarcopenia. Nevertheless, dietary diaries and 24 h urinary collections did not show any difference in nutrients intake between the two groups. However, self-reporting of nutrients intake by means of food diaries may have introduced some bias [20,21,22,23,24,25,26]. In particular, in the elderly, energy under-reporting was found more frequently in overweight individuals [28]. In consideration of the fact that in our cohort non-sarcopenic individuals were predominantly overweight, it is possible that they had a substantially higher rate of under-reporting of energy intake.

On the other hand, since all patients were already on follow up; we cannot exclude that sarcopenic patients that had higher prevalence of PEW had been previously prescribed a hypercaloricand normo-proteic diet in order to correct PEW. This would explain why sarcopenic individuals were mostly in a normo-proteic diet despite having a worse renal function.

Sarcopenic patients had worse physical performance as indicated by Short Physical Performance Battery and by IADL scores. In patients with CKD, sarcopenia and physical inactivity may progress together and are independent predictors of mortality [29].

Consistent with what was previously described [5], in sarcopenic individuals, the reduction of physical performance was mainly limited to the gait speed test that is dependent on reduced muscular performance but is also influenced by overall cardiovascular function. In a recent study in CKD patients, Lai and colleagues found that sarcopenia was associated with vascular dysfunction and overall increased cardiovascular risk. In our study, we did not specifically gather data on cardiovascular risk but sarcopenic and non-sarcopenic patients did not show any difference regarding the proportion of diabetes and previous cardiovascular events as well as they did not have any difference in lipid profile.

In our cohort, sarcopenic patients had a higher prevalence of depression. This may be explained by the fact that sarcopenia and depression are associated to some common features such as reduced physical activity and inadequate dietary intake [9,30]. Recently, Lai and colleagues found that sarcopenic CKD patients had a higher prevalence of depression with respect to non-sarcopenic ones. These results confirm what was previously found in not-CKD individuals. A study conducted in a population of older Chinese men demonstrated that muscle mass was inversely associated with the severity of depression [31]. Likewise, another study conducted among older individuals found that depression was independently correlated to reduced muscle mass [32].

In older patients, the immunoregulatory system is often unbalanced toward a pro-inflammatory state [33,34], and the accumulation of proinflammatory cytokines such as IL-6 and TNFα is associated with the development of sarcopenia [35,36,37]. Likewise, also in patients affected by CKD, the progressive reduction of renal function induces an accumulation of inflammatory cytokines that could contribute to the development of sarcopenia. However, in CKD patients, the association between inflammatory mediators and the development of sarcopenia was not consistently observed [5,38].

In our study, CKD patients had higher concentrations of several proinflammatory cytokines. However, despite having considered a large pattern of pro-inflammatory and anti-inflammatory cytokines, we did not observe any difference in systemic inflammation between sarcopenic and non-sarcopenic individuals. It is therefore plausible that in a condition such as CKD, where there is a generalized activation of systemic inflammatory response, inflammation per se is not sufficient to induce the development of sarcopenia. Some authors refer to muscle wasting in CKD as a process associated with specific intramuscular, but not systemic, inflammation [39]. Therefore, we hypothesize that individual susceptibility of muscular tissue to systemic inflammation might help to explain the inconsistent results that were reported by the studies conducted in CKD patients.

Our study has some limitations. First of all, the small sample size may have limited the observation of some associations between sarcopenia and possible related factors. Furthermore, the cross-sectional design does not take into account the time of exposure to the potential risk factors; therefore, we cannot prove any causality in the associations that were observed. However, we applied stringent selection criteria that allowed us to limit the possible confounding factors. Indeed, we excluded the majority of the conditions that may have influenced the inflammatory status and we analyzed a vast spectrum of pro- and anti-inflammatory pathways that depict a rather complete picture of systemic inflammatory status.

## 5. Conclusions

In conclusion, we highlighted several modifiable conditions that are associated with sarcopenia in older CKD individuals. In particular, we observed a significant association of sarcopenia with MIS and PEW syndrome. We found also that sarcopenia was associated with unintentional weight loss and with reduced fat mass at the bio-impedentiometry, conditions that may both depend on an insufficient alimentary intake.

Overall, we believe that our results represent a valuable basis to define which clinical variables, lifestyle habits and molecular biomarkers should be evaluated in prospective trials designed to understand the pathophysiology of sarcopenia in CKD patients.

## Figures and Tables

**Table 1:** Cohort characteristics.

Variables	Overall Cohort (*n* = 113)	No Sarcopenia (*n* = 86)	Sarcopenia (*n* = 27)	*p*
Age (years)	80 ± 6	80 ± 6	79 ± 6	0.6
Male	77 (68%)	56 (65%)	21 (78%)	0.28
Diabetes	61 (54%)	48 (56%)	13 (48%)	0.41
Previous cardiovascular events	61 (54%)	43 (53%)	18 (67)	0.12
eGFR * (mL/min/1.73 m^2^)	27 ± 6	28 ± 5	25 ± 3	0.25
Creatinine	2.5 ± 1.2	2.6 ± 1.0	2.8 ± 1.3	0.29
Creatinine Clearance (mL/min/1.73 m^2^)	27 ± 14	28 ± 15	19 ± 12	0.01
*Criteria for sarcopenia*				
Reduced handgrip strength (%)	63	52	100	<0.0001
Reduced MAMC ** (%)	34	13	100	<0.0001
Reduced Gait Speed Test (%)	69	63	88	0.028
*Physical performance*				
PAS ***	7 ± 5	7 ± 5	4 ± 6	0.0014
SPPB ****	7 ± 4	8 ± 4	6 ± 3.75	0.0008
IADL scale *****	5.0 ± 1.0	5.0 ± 1.0	5 ± 1.8	0.016
Frail patients (%) ******	50 (45%)	33 (38%)	17 (63%)	0.023
Depression (%)	33	67	32	0.16
Depression score	9.1 ± 6.0	8.3 ± 5.5	11.8 ± 7.1	0.008

* eGFR: estimated glomerular filtration rate; ** MAMC: mid arm muscle circumference. *** PAS: physical activity scale; **** SPPB: short physical performance battery; ***** IADL: instrumental activities of daily life (Lawton’s scale), ****** According to Frailty phenotype.

**Table 2:** Biochemical nutritional parameters and estimated nutritional intake.

Variables	Overall Cohort (*n* = 113)	No Sarcopenia (*n* = 86)	Sarcopenia (*n* = 27)	*p*
Prescribed hypoproteic diet, *n* (%)	36 (33%)	33 (38%)	3 (11%)	0.0065
*Serum parameters*				
Albumin (g/dL)	4.1 ± 0.4	4.0 ± 0.3	4.1 ± 0.4	0.73
Prealbumin (mg/dL)	28 ± 5	28 ± 5	28 ± 6	0.74
Total Cholesterol (mg/dL)	159 ± 42	157 ± 39	174 ± 52	0.25
HDL (mg/dL)	52 ± 18	51 ± 18	56 ± 15	0.19
LDL (mg/dL)	89 ± 30	87 ± 29	93 ± 33	0.37
Triglycerides (mg/dL)	128 ± 55	129 ± 57	123 ± 51	0.6
Transferrin (mg/dL)	229 ± 37	231 ± 40	228 ± 40	0.68
Transferrin saturation, %	22 ± 11	23 ± 9	22 ± 8	0.61
Potassium (mmol/L)	4.7 ± 0.5	4.6 ± 0.4	4.7 ± 0.5	0.19
Phosphorous (mg/dL)	3.6 ± 5.6	3.5 ± 0.6	3.8 ± 0.6	0.07
Uric Acid (mg/dL)	6.1 ± 1.4	6.1 ± 1.3	6.2 ± 1.8	0.8
*Vitamin D status*				
Vitamin D 25 OH (ng/mL)	27 ± 21	29 ± 17	29 ± 17	0.96
<15 ng/mL, %	19(22/113)	17 (15/86)	25 (7/27)	0.36
15–30 ng/mL, %	38(44/113)	44 (38/86)	25 (7/27)	0.07
>30 ng/mL, %	38(44/113)	36 (31/86)	48 (13/27)	0.30
Calcifediol supplementation, %	69 (79/113)	70 (61/86)	66 (18/27)	0.49
*24 h urine collection*				
Urinary urea (mg/24 h)	15,450 ± 8050	16,400 ± 8832	13,600 ± 6022	0.02
Urinary urea/BMI ((mg/24 h)/(kg/m^2^))	573 ± 192	571 ± 186	576 ± 217	0.97
Urinary urea/Urinary creatinine	17.9 ± 5.1	17.3 ± 4.6	19.1 ± 7.2	0.075
nPCR (g/kg/24 h)	0.73 ± 0.28	0.72 ± 0.30	0.74 ± 0.21	0.75
Urinary creatinine (mg/24 h)	898 ± 316	920 ± 320	713 ± 274	0.0034
Urinary creatinine/BMI (mg/(kg/m^2^))	31.4 ± 11.6	32.2 ± 11.9	28.8 ± 10.6	0.19
Urinary creatinine/height (mg/m)	532 ± 184	565 ± 183	442 ± 158	0.0024
Urinary Phosphorous (mg/24h)	503 ± 194	512 ± 197	381 ± 146	0.0025
*Estimated nutritional intake*				
Calories (Kcal)	1538 ± 414	1519 ± 436	1572 ± 375	0.60
Kcal/weigh (Kcal/Kg)	21 ± 9	20 ± 9	24 ± 8	0.0058
Proteins (%)	15 ± 5	15 ± 5	15 ± 5.75	0.33
Carbohydrates (%)	45 ± 9	45 ± 9	45 ± 7	0.93
Lipids (%)	39 ± 8	40 ± 8	39 ± 7	0.73

nPCR, normalized Protein Catabolic Rate; HDL, High density lipoprotein; LDL, Low Density Lipoprotein; BMI, body mass index.

**Table 3:** Body composition and nutritional indices.

Nutritional Variables	Overall Cohort (*n* = 113)	No Sarcopenia (*n* = 86)	Sarcopenia (*n* = 27)	*p*
BMI (kg/m^2^)	27.5 ± 5.6	28.4 ± 5.5	24.8 ± 3.0	<0.0001
*Body composition (bio-impedentiometry)*				
OH (L)	1.3 ± 1.7	1.2 ± 1.8	1.4 ± 1.4	0.62
LTI (kg/m^2^)	13.5 ± 2.9	13.5 ± 3.1	13.3 ± 2.9	0.75
Lean tissue proportion (%)	49 ± 12	48 ± 12	51 ± 11	0.30
FTI (kg/m^2^)	13.7 ± 4.9	14.4 ± 5.1	11.6 ± 3.9	0.023
Fat tissue proportion (%)	35 ± 9	36 ± 9	33 ± 8	0.20
Lean/fat tissue ratio	1.6 ± 0.98	1.5 ± 0.9	1.8 ± 1.2	0.34
MIS	5 ± 5	4.5 ± 4	6 ± 6.5	0.09
PEW	32 (25%)	18 (20%)	14 (52%)	0.0008
*ISRNM criteria for PEW*				
Serum albumin < 3.8 g/dL	28 (25%)	21 (24%)	7 (26%)	0.63
Serum prealbumin < 30 mg/dL	59 (56%)	43 (50%)	16 (59%)	0.12
Serum total cholesterol < 100 mg/dL	1 (0%)	1 (1%)	0 (0%)	0.60
BMI < 23 kg/m^2^	14 (12%)	11 (12%)	3 (11%)	0.90
Unintentional weight loss over time ^1^	19 (17%)	14 (16%)	5 (18%)	0.50
Total body fat percentage < 10%	2 (2%)	2 (2%)	0 (0%)	0.45
Muscle wasting ^2^	5 (4%)	4 (5%)	1 (4%)	0.92
Unintentional low DPI ^3^	16 (14%)	14 (16%)	2 (7%)	0.33
Unintentional low DEI ^4^	49 (51%)	40 (46%)	9 (33%)	0.42

BMI: body mass index; OH, over-hydration; LTI, lean tissue index. LTM, lean tissue mass. FTI, fat tissue index. MIS, Malnutrition Inflammation score. PEW, Protein Energy Wasting. ISRNM, International Society of Renal Nutrition and Metabolism. MAMC, mid-arm muscle circumference. DPI, daily protein intake. DEI, daily energy intake. ^1^ Five percent over 3 months or 10% over 6 months’; ^2^ educed muscle mass 5% over 3 months or 10% over 6 months; ^3^ <0.60 g kg^−1^ day^−1^ for at least 2 months; ^4^ <25 kcal kg^−1^ day^−1^ for at least 2 months.

**Table 4:** Cytokines in CKD patients and healthy controls.

Cytokines	Healthy Controls (*n* = 15)	CKD (*n* = 109)	*p*
TNF alpha (pg/mL)	7.1 (1.5–20.5)	14.6 (2.4–42.5)	0.014
IL-12p70 (pg/mL)	0 (0–1.7)	0.7 (0–19)	0.002
MCP-1 (pg/mL)	254 (199–486)	407 (21–886)	0.002
Fetuin A (g/L)	0.41 (0.18–0.84)	0.31 (0.12–0.76)	0.010
IL-6 (pg/mL)	1.1 (0–17.3)	3.4 (0–12.9)	0.066
IL-10 (pg/mL)	5 (1–18)	2 (0–740)	0.464
IL-17 (pg/mL)	1.9 (0.5–6.8)	1.5 (0–17.6)	0.314

**Table 5:** Inflammatory markers in non-sarcopenic and sarcopenic patients.

Inflammatory Markers	No Sarcopenia (*n* = 86)	Sarcopenia (*n* = 27)	*p*
TNFα (pg/mL)	13.6 (2.9–48.8)	14.6 (2.4–31.4)	0.981
IL-12p70 (pg/mL)	0.7 (0–15.9)	0.6 (0–19.3)	0.842
MCP-1 (pg/mL)	408 (222–886)	429 (21–796)	0.543
Fetuin A (ng/mL)	0.29 (0.12–0.76)	0.29 (0.22–0.52)	0.445
IL-6 (pg/mL)	3.1 (0–12.9)	4.1 (0–11.7)	0.252
IL-10 (pg/mL)	2 (0–740)	1.4 (0.2–295)	0.467
IL-17 (pg/mL)	0 (0–8.3)	0 (0–2.9)	0.303
CRP (mg/dL)	0.24 (0.30–3.65)	0.20 (0.05–4.68)	0.823

CRP: C reactive protein.
